# Assessment of *pTERT* Subtypes of Glioblastoma by Quantitative FLAIR Analysis Based on Fusing MRI Images

**DOI:** 10.1002/brb3.71284

**Published:** 2026-03-12

**Authors:** Bocong Gao, Guanmin Quan, Yawu Liu, Tao Yuan

**Affiliations:** ^1^ Department of Medical Imaging The Second Hospital of Hebei Medical University Shijiazhuang China; ^2^ Department of Clinical Radiology Kuopio University Hospital Kuopio Finland; ^3^ Department of Neurology University of Eastern Finland Kuopio Finland

**Keywords:** FLAIR metrics, fused imaging, glioblastoma, quantitative analysis, *TERT* promoter

## Abstract

**Background:**

Mutations in the telomerase reverse transcriptase promoter (*pTERT*) are important molecular markers in glioblastoma (GBM). Although several imaging‐based approaches have attempted to predict *pTERT* mutation status preoperatively, the value of quantitative metrics extracted from lesion subregions remains unclear. This study investigated whether quantitative FLAIR metrics derived from contrast‐enhanced T1‐weighted imaging (CE T1WI)–FLAIR fused images contribute to the differentiation of *pTERT* subtypes in GBM.

**Methods:**

MRI and clinical data from 135 GBM patients, 94 with *pTERT*‐mutant (*pTERTm*) and 41 with *pTERT*‐wild‐type (*pTERTw*) tumors, were retrospectively analyzed. Patients were randomly assigned to training and validation cohorts in a 7:3 ratio. Clinical characteristics and conventional MRI variables were compared between *pTERTm* and *pTERTw* groups. FLAIR signal intensity (SI) metrics were measured in three subregions on CE T1WI–FLAIR fused images: the enhancement region, the edema region (non‐enhancing), and the whole lesion (enhancement + edema). Significant variables identified by logistic regression were incorporated into clinical, MRI, and combined predictive models. Model performance was internally evaluated using leave‐one‐out cross‐validation (LOOCV) in the training cohort and externally assessed in the validation cohort.

**Results:**

Significant differences between *pTERTm* and *pTERTw* groups were observed in age, FLAIR SI standard deviation (FLAIR_SD_) and relative FLAIR SI (rFLAIR) of the edema region, and FLAIR_SD_ of the enhancement region (all *p* < 0.05). Logistic regression identified older age (> 42.5 years; OR = 1.09; *p* = 0.002), higher FLAIR_SD_ in the enhancement region (> 62.45; OR = 1.01; *p* = 0.027), and higher rFLAIR in the edema region (> 1.706; OR = 7.49; *p* = 0.025) as independent predictors of *pTERTm*. In the training cohort, the combined model achieved an area under the ROC curve (AUC) of 0.833, outperforming the clinical model (0.675) and MRI‐based model (enhancement‐region FLAIR_SD_: 0.737; edema‐region rFLAIR: 0.699). The combined model achieved the highest predictive performance, with LOOCV in the training cohort yielding a mean AUC of 0.784 (95% CI: 0.672–0.895) and external validation showing an AUC of 0.667 (95% CI: 0.466–0.867).

**Conclusions:**

Quantitative FLAIR metrics extracted from subregions on CE T1WI–FLAIR fused images differ significantly between *pTERTm* and *pTERTw* GBM. Subregional quantitative analysis may therefore contribute to noninvasive preoperative prediction of *pTERT* mutation status.

## Graphical abstract

Assessment of pTERT subtypes of glioblastoma by quantitative FLAIR analysis based on fusing MRI images, Participants, Methods, Results, Glioblastoma patients, Segmented to 3 subregions Investigated the significant metrics of subregions and other clinical‐imaging factors Prediction models of pTERT Tested in validation cohorts pTERTm, pTERTw, **Conclusion**: Quantitative metrics extracted from subregional segmentation of CE T1WI‐ FLAIRfusion images provided incremental predictionof pTERTm in GBM patients.: Quantitative metrics extracted from subregional segmentation of CE T1WI‐ FLAIRfusion images provided incremental predictionof pTERTm in GBM patients.

Prediction for pTERT	Cutoffvalue	Accuracy	PPV	NPV	AUC(95%CI)
Age(years)	42.5	0.800	0.800	0.429	0.675 (0.539‐0.811)
CEFLAIR_SD_	62.452	0.811	0.802	0.429	0.737 (0.618‐0.855)
NCE rFLAIR	1.706	0.642	0.884	0.821	0.699 (0.588‐0.810)
All	‐	0.811	0.889	0.750	0.833 (0.735‐0.940)

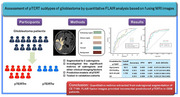

## 1 Introduction

Telomerase reverse transcriptase promoter (*pTERT*) mutation is an important molecular marker in glioblastoma (GBM) and is associated with poor response to temozolomide (TMZ) chemotherapy, abnormal tumor microenvironment characteristics, and unfavorable survival outcomes (Kurokawa et al. 2022; Murugan et al. 2025; Tang et al. 2023). Although histopathological analysis of surgical or biopsy specimens remains the gold standard for determining *pTERT* status, such testing is time‐consuming and not routinely performed in most medical centers. Delayed diagnosis or limited access to molecular testing may hinder timely postoperative salvage therapy, especially given that tumor recurrence can occur as early as 27 days after resection (Behling et al. 2022). Therefore, accurate preoperative identification of *pTERTm* is clinically meaningful and may influence treatment strategies, including telomerase‐targeted therapies.

Advanced MRI techniques such as diffusion‐weighted imaging (DWI), dynamic contrast‐enhanced perfusion‐weighted imaging (DCE‐PWI), and artificial intelligence‐based methods have shown promise for predicting *pTERT* genetic status (Chen et al. 2023; Behling et al. 2022; Liu et al. 2022; Park et al. 2020; Zhang et al. 2020; Zhang et al. 2023). However, the complexity of these approaches and the variability in image post‐processing limit their practicality in routine clinical settings. Conventional MRI (cMRI) features, including tumor location, size, and necrosis volume, are fundamental to preoperative assessment but have limited diagnostic value for *pTERT* genetic profiling (Ivanidze et al. 2019; Yamashita et al. 2019). Radiomics‐based methods can assist in *pTERT* prediction, but their reliance on specialized software and complex workflow restricts widespread clinical adoption. Thus, clinically accessible cMRI‐based imaging markers for predicting *pTERT* mutation status are needed.

Most cMRI studies focusing on *pTERT* prediction rely on measurements of tumor size or extent on CE T1WI and FLAIR imaging, but lack quantitative assessment of signal intensity (SI) changes. Quantitative FLAIR metrics in residual cavities and surrounding regions have been used to predict GBM recurrence (Gao et al. 2024). In preoperative settings, FLAIR hyperintense regions extending beyond enhancement may contain sparse tumor infiltration. These regions, whether enhancing or non‐enhancing, should be analyzed separately, given that distinct *pTERT* mutation types may correspond to different pathological characteristics (Zhang, Ouyan, et al. 2024).

We hypothesized that quantitative FLAIR metrics in different subregions may reflect underlying pathological differences related to *pTERT* mutation status. To test this hypothesis, we evaluated the predictive value of quantitative FLAIR features derived from CE T1WI–FLAIR fused imaging.

## 2 Materials and Methods

### 2.1 Patient Selection

This retrospective study was approved by the ethics committee of The Second Hospital of Hebei Medical University (2024‐R186). The requirement for informed consent was waived due to the retrospective design and the use of anonymized data. Consecutive patients with GBM treated at our hospital between December 2019 and December 2023 were retrospectively reviewed. Patients were included if they met the following criteria: (1) age > 18 years, (2) pathologically confirmed GBM with available *pTERT* genetic profiling, and (3) preoperative conventional non‐contrast and contrast‐enhanced MRI performed within 1 week prior to surgery. Exclusion criteria were (1) presence of other coexisting central nervous system tumors and (2) MRI images of insufficient quality for evaluation.

GBM diagnosis followed the 2021 *WHO Classification of Tumors of the Central Nervous System* (fifth edition) (Kurokawa et al. 2022). Based on polymerase chain reaction results, *pTERT* genetic status was categorized as mutant (*pTERTm*) or wild type (*pTERTw*).

### 2.2 Trial Registration

Not applicable. This study is a retrospective analysis of previously acquired clinical and MRI data and did not involve any prospective patient enrollment or intervention.

### 2.3 MRI Acquisition and Analysis

All MRI examinations were performed on a GE Architect 3.0 T scanner using a 48‐channel phased‐array head coil. Preoperative imaging included a 3D gradient‐echo T1‐weighted sequence and axial or 3D FLAIR sequences. Post‐contrast imaging was acquired following intravenous administration of gadolinium (Gd‐DTPA; 0.1 mL/kg at 3.0 mL/s), followed by a 20‐mL saline flush.

cMRI variables assessed included tumor location (frontal, temporal, or other lobes), corpus callosum invasion, and ependymal invasion (Yamashita et al. 2019).

FLAIR and CE T1WI images were fused using 3D Slicer (version 5.2.1, https://www.slicer.org/). On the fused images, FLAIR‐hyperintense lesions were segmented into three subregions: enhancement region, non‐enhancing edema region, and whole lesion (enhancement + edema). Measurements were performed on the slice showing the largest area of enhancement. Regions of interest (ROIs) with similar shape and size (15–20 mm^2^) were placed in the three lesion subregions and background tissue. Quantitative FLAIR metrics included: minimal SI (FLAIR_min_), mean SI (FLAIR_mean_), median SI (FLAIR_median_), standard deviation of SI (FLAIR_SD_), and relative FLAIR SI (rFLAIR = (mean SI_subregion − mean SI_background)/(mean SI_contralateral − mean SI_background)) (Yuan et al. 2022).

Functional MRI metrics were analyzed on a GE AW 4.7 workstation. Three ROIs (15–20 mm^2^) were placed in the prominent enhancement region, avoiding vessels, calcification, necrosis, and hemorrhage. Choline (Cho) and creatine (Cr) ratios were recorded. For DWI, ADC values were measured in the enhancement region; the minimum ADC value was recorded, and a corresponding ROI was placed in contralateral normal parenchyma to calculate relative ADC (rADC = ADC_lesion_/ADC_contralateral_).

All imaging evaluations were independently performed by two radiologists with 4 and 15 years of experience, respectively; discrepancies were resolved by a third neuroradiologist with 32 years of experience.

### 2.4 Statistical Analysis

Statistical analyses were conducted using SPSS (version 17.2.0; SAS Institute Inc., Cary, North Carolina, USA) and MSTATA (https://www.mstata.com/). Categorical variables were expressed as percentages and compared using *χ^2^* tests. Continuous variables were expressed as mean ± standard deviation and compared using unpaired *t*‐tests for normally distributed variables, or median (interquartile range) and Mann–Whitney *U* tests for non‐normal distributions.

Univariate and multivariate logistic regression analyses were performed to identify significant predictors of *pTERTm*. Variables with *p* < 0.05 in univariate analysis were entered into the development of the clinical, MRI, and combined predictive models. Diagnostic performance, including accuracy, sensitivity, specificity, positive predictive value (PPV), negative predictive value (NPV), and the area under the ROC curve (AUC), was assessed using ROC analysis. Variables that remained significant (*p* < 0.05) in the multivariate model were used to construct a nomogram for estimating the probability of *pTERT* mutation.

To internally assess model robustness, leave‐one‐out cross‐validation (LOOCV) was performed within the training cohort. The final multivariate model was subsequently applied to the independent validation cohort to evaluate its generalization performance.

Interobserver reliability was assessed using the intraclass correlation coefficient (ICC), with ICC > 0.75 indicating good agreement. Statistical significance was defined as *p* < 0.05.

## 3 Results

### 3.1 General Information

A total of 148 patients with GBM were screened, and 13 were excluded due to coexisting CNS tumors (*n* = 3) or poor imaging quality (*n* = 10). Thus, 135 patients were included in the final analysis; 76 males and 59 females, aged 23–78 years (mean age 56 years). Among them, 94 had *pTERTm* and 41 had *pTERTw*.

Patients were randomly assigned to the training cohort (*n* = 95) and validation cohort (*n* = 40) at a 7:3 ratio. The training cohort included 67 *pTERTm* and 28 *pTERTw* patients, whereas the validation cohort included 27 *pTERTm* and 13 *pTERTw* patients. No significant differences in clinical or imaging variables were observed between the two cohorts (all *p* > 0.05) (Table 1).

**TABLE 1:** Characteristics of patients with GBM in the training and validation cohorts (*n* = 135).

Characteristics	Training cohort (*n* = 95)	Validation cohort (*n* = 40)	*p* value
**Clinical characteristics**			
Age (years), median (IQR)	59 (50, 65)	57 (50, 66)	0.666
Gender, male, *n*%	52 (54.7%)	24 (60.0%)	0.573
**cMRI characteristics**			
Tumor location, temporal lobe, *n*%	41 (43.2%)	11 (27.5%)	0.200
SVZ involvement, *n*%	65 (68.4%)	22 (55.0%)	0.137
Corpus callosum involvement, *n*%	22 (23.2%)	12 (30.0%)	0.403
**MRI quantitative characteristics**			
NCE			
Area (cm^2^), median (IQR)	6.0 (2.9, 9.3)	5.6 (3.5, 11.4)	0.356
FLAIR_min_, median (IQR)	525 (401, 680)	476 (378, 627)	0.231
FLAIR_max_, median (IQR)	833 (643, 944)	825 (592, 911)	0.549
FLAIR_mean_, median (IQR)	801 (637, 900)	796 (577, 877)	0.454
FLAIR_median_, median (IQR)	815 (640, 927)	811 (587, 903)	0.465
FLAIR_SD_, median (IQR)	90 (68, 128)	93 (70, 127)	0.883
rFLAIR, median (IQR)	1.66 (1.48, 2.08)	1.61 (1.36, 2.15)	0.545
CE			
Area (cm^2^), median (IQR)	7.4 (4.3, 11.3)	8.2 (4.5, 10.9)	0.594
FLAIR_min_, median (IQR)	347 (204, 486)	285 (191, 380)	0.067
FLAIR_max_, median (IQR)	741 (604, 872)	712 (515, 777)	0.102
FLAIR_mean_, median (IQR)	709 (586, 841)	670 (495, 756)	0.163
FLAIR_median_, median (IQR)	720 (606, 833)	693 (502, 771)	0.225
FLAIR_SD_, median (IQR)	111 (77, 147)	114 (76, 147)	0.944
rFLAIR, median (IQR)	1.56 (1.18, 1.91)	1.33 (1.16, 1.78)	0.277
NCE + CE			
Area (cm^2^), median (IQR)	15 (9, 19)	16 (10, 22)	0.495
FLAIR_min_, median (IQR)	347 (204, 475)	272 (191, 326)	0.052
FLAIR_max_, median (IQR)	784 (661, 909)	770 (575, 836)	0.173
FLAIR_mean_, median (IQR)	749 (647, 866)	729 (566, 807)	0.181
FLAIR_median_, median (IQR)	770 (640, 883)	761 (564, 840)	0.326
FLAIR_SD_, median (IQR)	118 (88, 149)	112 (72, 167)	0.709
rFLAIR, median (IQR)	1.56 (1.32, 2.06)	1.52 (1.22, 1.89)	0.295
MRS (*n* = 106)			
Cho/Cr, median (IQR)	3.40 (2.27, 5.02)	2.88 (1.95, 3.46)	0.066
DWI/DTI (*n* = 90)			
rADC, median (IQR)	0.96 (0.83, 1.11)	0.95 (0.89, 1.13)	0.377

Abbreviations: CE, enhancing region; cMRI, coventional MRI; GBM, glioblastoma; IQR, interquartile range; NCE, non‐enhancing region; SVZ, subventricular zone.

### 3.2 Comparison Between *pTERTm* and *pTERTw* Groups

Compared with *pTERT*w patients, those with *pTERTm* were older (median 62 vs. 54 years, *p* = 0.008), had higher FLAIR_SD_ (median 97 vs. 75, *p* = 0.003) and rFLAIR (median 1.77 vs. 1.53, *p* = 0.002) in the edema region, and higher FLAIR_SD_ in the enhancement region (median 121 vs. 83, *p* < 0.001).

**FIGURE 1:**
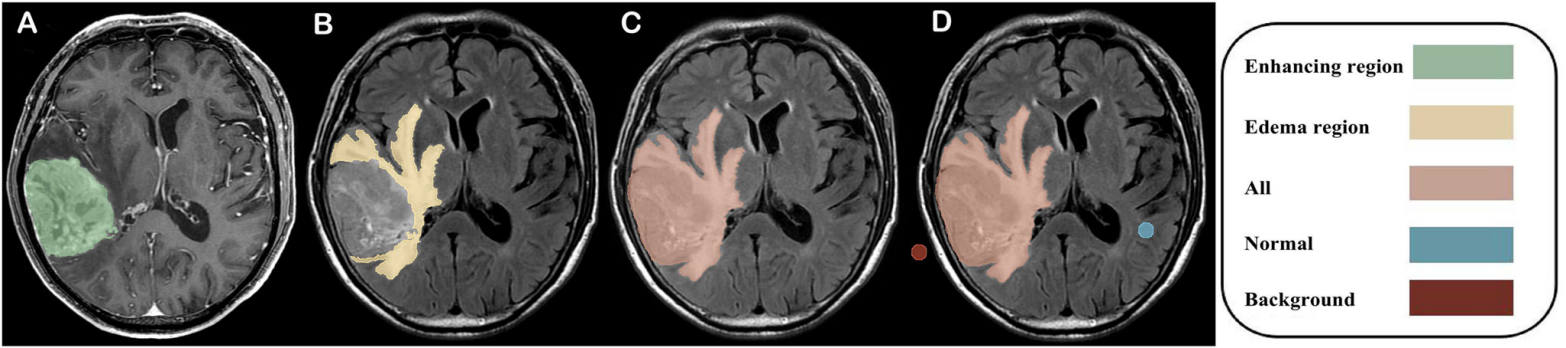
Representative case of *pTERTm* GBM. A 74‐year‐old male patient presented with a glioblastoma in the left parietal lobe. (A) The standard deviation of FLAIR signal intensity (FLAIR_SD_) in the enhancement region was 152.37. (B) In the edema region, FLAIR_SD_ and relative FLAIR (rFLAIR) were 148.30 and 2.06, respectively. (C, D) The FLAIR signal intensities of the whole lesion, contralateral normal region, and background were 355.81, 193.73, and 3.49, respectively. Nomogram analysis yielded a total score of 80, corresponding to a predicted probability of *pTERT* mutation of 0.97.

**FIGURE 2:**
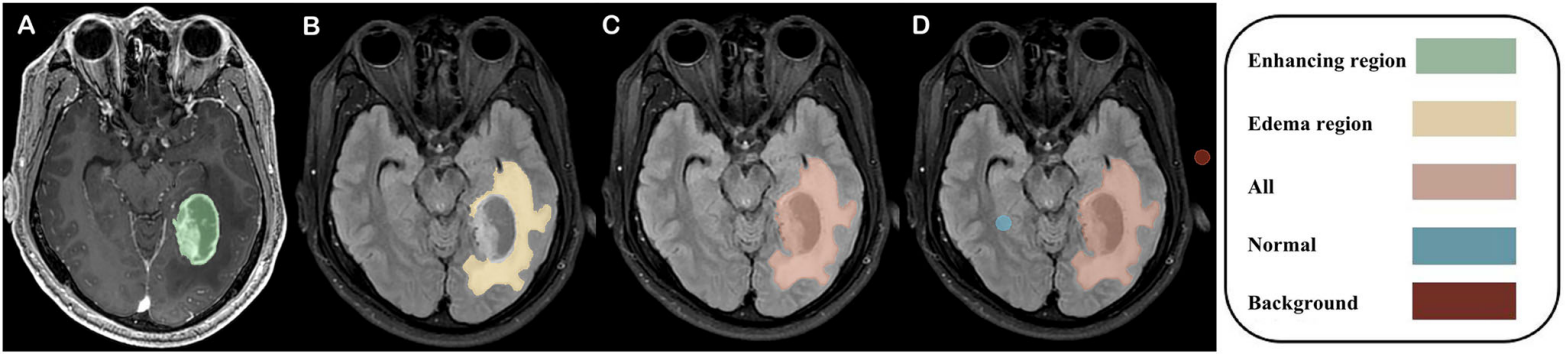
Representative case of *pTERTw* GBM. A 32‐year‐old male patient presented with a glioblastoma in the right temporal lobe. (A) The FLAIR_SD_ in the enhancement region was 47.39. (B) In the edema region, FLAIR_SD_ and rFLAIR were 69.29 and 1.54, respectively. (C, D) The FLAIR signal intensities of the whole lesion, contralateral normal region, and background were 867.37, 621.89, and 11.19, respectively. Nomogram analysis yielded a total score of 26, corresponding to a predicted probability of *pTERT* mutation of 0.06.

No significant differences were observed in gender, tumor location, corpus callosum or ependymal invasion, edema extent, or other FLAIR metrics (Table 2). Representative cases of *pTERTm* and *pTERTw* are shown in Figures 1 and 2.

**TABLE 2:** Baseline characteristics of *pTERTw* and *pTERTm* GBM patients of training cohort (*n* = 95).

Characteristics	*pTERTw* (*n* = 28)	*pTERTm* (*n* = 67)	*p* value
**Clinical characteristics**			
Age (years), median (IQR)	54 (38, 63)	62 (55, 65)	0.008
Gender, male, *n*%	13 (46%)	39 (58%)	0.293
**cMRI characteristics**			
Tumor location, temporal lobe, *n*%	13 (46%)	28 (42%)	0.543
SVZ involvement, *n*%	19 (68%)	46 (69%)	0.939
Corpus callosum involvement, *n*%	5 (18%)	17 (25%)	0.429
**MRI quantitative characteristics**			
NCE			
Area (cm^2^), median (IQR)	5.7 (3.1, 8.2)	6.1 (2.8, 9.4)	> 0.999
FLAIR_min_, median (IQR)	508 (364, 661)	532 (402, 682)	0.656
FLAIR_max_, median (IQR)	806 (544, 923)	844 (724, 945)	0.210
FLAIR_mean_, median (IQR)	771 (484, 877)	820 (702, 907)	0.114
FLAIR_median_, median (IQR)	788 (508, 920)	844 (707, 934)	0.129
FLAIR_SD_, median (IQR)	75 (45, 99)	97 (79, 132)	0.003
rFLAIR, median (IQR)	1.53 (1.39, 1.66)	1.77 (1.49, 2.17)	0.002
CE			
Area (cm^2^), median (IQR)	6.7 (4.5, 11.3)	7.6 (4.3, 11.2)	0.732
FLAIR_min_, median (IQR)	399 (252, 483)	334 (195, 486)	0.535
FLAIR_max_, median (IQR)	722 (508, 834)	748 (641, 877)	0.174
FLAIR_mean_, median (IQR)	702 (427, 746)	730 (602, 848)	0.109
FLAIR_median_, median (IQR)	711 (424, 760)	740 (618, 843)	0.143
FLAIR_SD_, median (IQR)	83 (46, 112)	121 (90, 156)	< 0.001
rFLAIR, median (IQR)	1.41 (1.10, 1.79)	1.62 (1.23, 2.00)	0.128
NCE + CE			
Area (cm^2^), median (IQR)	16 (9, 19)	15 (9, 19)	0.864
FLAIR_min_, median (IQR)	393 (252, 465)	334 (189, 476)	0.433
FLAIR_max_, median (IQR)	762 (558, 885)	802 (691, 926)	0.245
FLAIR_mean_, median (IQR)	734 (438, 843)	763 (673, 883)	0.162
FLAIR_median_, median (IQR)	754 (436, 860)	799 (662, 883)	0.177
FLAIR_SD_, median (IQR)	114 (68, 138)	124 (95, 149)	0.187
rFLAIR, median (IQR)	1.47 (1.22, 1.86)	1.61 (1.37, 2.08)	0.248

Abbreviations: CE, enhancing region; cMRI, coventional MRI; GBM, glioblastoma; IQR, interquartile range; NCE, non‐enhancing region; *pTERTm*, mutation‐type *pTERT; pTERTw*, wild‐type *pTERT*; SVZ, subventricular zone.

### 3.3 Modeling and Evaluation

Univariate logistic regression identified age (*p* = 0.001), FLAIR_SD_ (*p* = 0.008) and rFLAIR (*p* = 0.003) of the edema region, and FLAIR_SD_ of the enhancement region (*p* = 0.006) as significant predictors of *pTERTm* (Table 3).

**TABLE 3:** Univariate and multivariate analyses for *pTERTm* GBM patients of training cohort.

	Characteristics	OR	95% CI	*p* value
**Univariate analysis**	Age (> 42.5 years)	1.07	1.03–1.11	0.001
	NCE FLAIR_SD_ (> 109.51)	1.02	1.00–1.03	0.008
	CE FLAIR_SD_ (> 62.452)	1.02	1.00–1.03	0.006
	NCE rFLAIR (> 1.706)	8.63	2.04–36.53	0.003
**Multivariate analysis**	Age (> 42.5 years)	1.09	1.03–1.15	0.002
	CE FLAIR_SD_ (> 62.452)	1.01	1.00–1.03	0.027
	NCE rFLAIR (> 1.706)	7.49	1.28–43.72	0.025

Abbreviations: 95% CI, 95% confidence interval; CE, enhancing region; GBM, glioblastoma; NCE, non‐enhancing region; OR, odds ratio; *pTERTm*, mutation‐type *pTERT*.

In multivariate analysis, age (*p* = 0.002), FLAIR_SD_ of the edema region (*p* = 0.025), and FLAIR_SD_ of the enhancement region (*p* = 0.0276) remained independent predictors.

**FIGURE 3:**
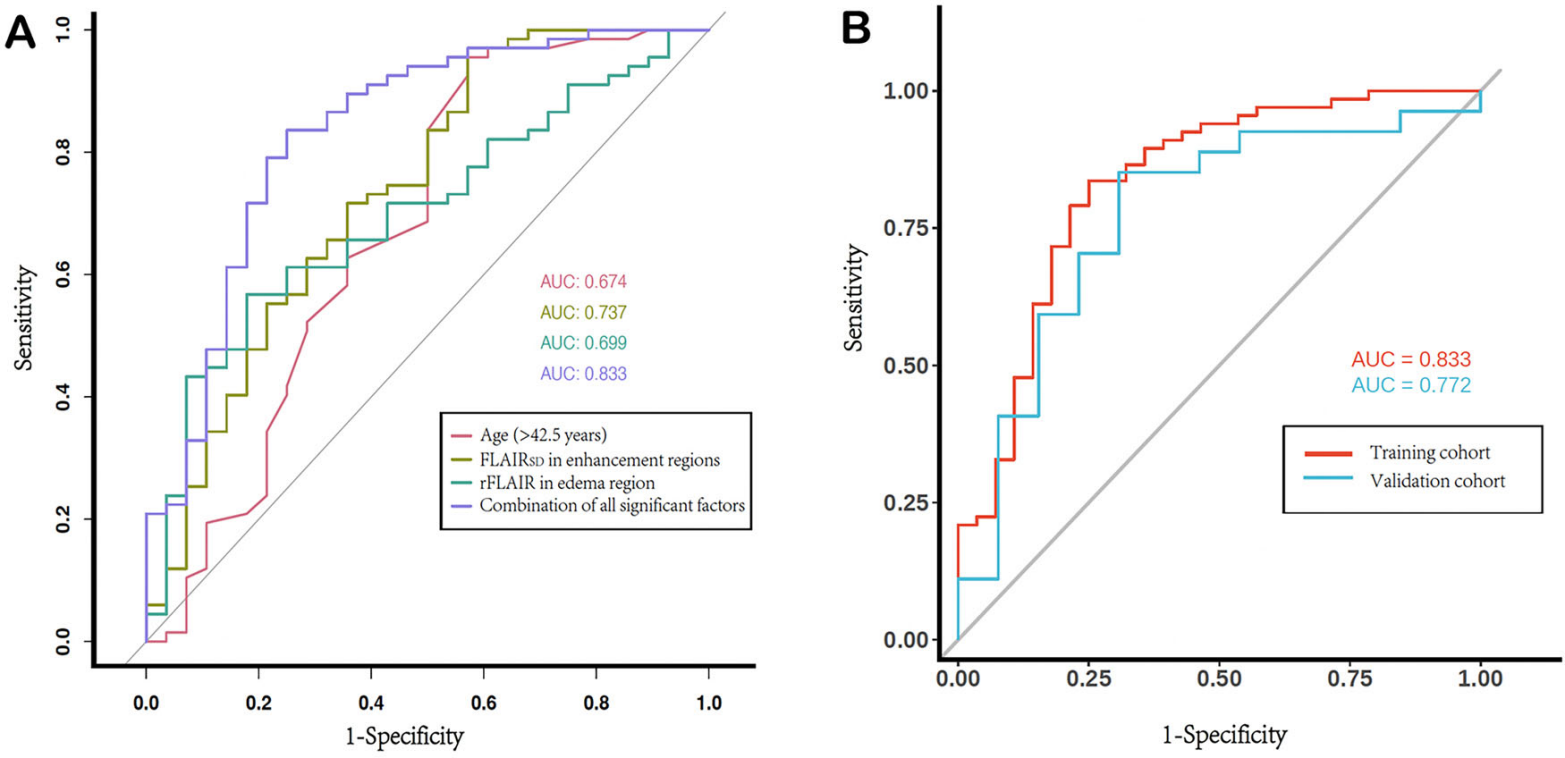
Receiver operating characteristic (ROC) analyses for predicting *pTERT* mutation in GBM. (A) ROC curves of individual predictive factors. (B) ROC curves of the combined predictive model in both the training cohort and the external validation cohort.

**FIGURE 4:**
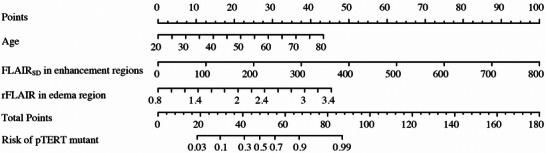
Nomograms for predicting *pTERT* mutation in GBM based on individual clinical and imaging factors.

These variables were incorporated into the clinical (age), MRI (FLAIR_SD_ of edema + FLAIR_SD_ of enhancement), and combined (all predictors) models (Table 4, Figure 3). In the training cohort, the clinical model yielded an AUC of 0.675 (95% CI, 0.539–0.811). The MRI subregional metrics demonstrated moderate diagnostic performance, with an AUC of 0.699 (95% CI, 0.588–0.810) for rFLAIR in the edema region and 0.737 (95% CI, 0.618–0.855) for the FLAIR_SD_ of the enhancement region. The combined model achieved the highest diagnostic accuracy, with an AUC of 0.833 (95% CI, 0.735–0.940). Cutoff values derived from ROC analysis indicated that an age threshold of 42.5 years, an rFLAIR value of 1.706 in the edema region, and a FLAIR_SD_ value of 62.45 in the enhancement region best distinguished *pTERTm* from *pTERTw*. Significant predictors, including age, CE‐region FLAIR_SD_, and NCE‐region rFLAIR (*p* < 0.05), were incorporated into nomograms to estimate the individualized probability of *pTERT* mutation (Figure 4).

**TABLE 4:** Efficacy of various factors for predicting *pTERTm* in GBM patients.

Characteristics	Cutoff value	Sensitivity	Specificity	Accuracy	PPV	NPV	AUC (95% CI)
Age (years)	42.5	0.955	0.429	0.800	0.800	0.429	0.675 (0.539–0.811)
CE FLAIR_SD_	62.452	0.970	0.429	0.811	0.802	0.429	0.737 (0.618–0.855)
NCE rFLAIR	1.706	0.567	0.821	0.642	0.884	0.821	0.699 (0.588–0.810)
Combined model (all)	—	0.836	0.750	0.811	0.889	0.750	0.833 (0.735–0.940)
Combined model LOOCV		0.791	0.714	0.768	0.869	0.588	0.784 (0.672–0.895)
Combined model validation		0.778	0.615	0.750	0.808	0.571	0.667 (0.466–0.867)

Abbreviations: 95% CI, 95% confidence interval; AUC, area under the curve; CE, enhancing region; GBM, glioblastoma; LOOCV, leave‐one‐out cross‐validation; NCE, non‐enhancing region; NPV, negative predictive value; PPV, positive predictive value; *pTERTm*, mutation‐type *pTERT*‌.

LOOCV performed on the training cohort demonstrated that the combined model achieved a mean AUC of 0.784 (95% CI, 0.672–0.895), indicating good internal stability. When applied to the independent validation cohort, the combined model yielded an AUC of 0.667 (95% CI, 0.466–0.867), supporting moderate generalization performance.

Interobserver reproducibility for the qualitative MRI features was excellent. The ICCs between the two radiologists were 0.958 for tumor location, 0.931 for corpus callosum involvement, and 0.941 for ependymal involvement (all *p* < 0.05), confirming the reliability of these imaging assessments.

## 4 Discussion

In the present study, logistic regression analyses identified age, FLAIR_SD_ of enhancement region, and rFLAIR of edema region as independent predictors of *pTERTm*. These variables were integrated into clinical, MRI, and combined predictive models. The combined model showed the best performance, achieving an AUC of 0.833 in the training cohort. LOOCV was performed to internally assess model stability, yielding a mean AUC of 0.784 (95% CI, 0.672–0.895), which demonstrates that the model is robust despite the relatively small sample size. Furthermore, external validation in the independent cohort produced an AUC of 0.667 (95% CI, 0.466–0.867), supporting the generalizability of the model. These results suggest that quantitative subregional FLAIR metrics combined with clinical factors can provide reliable preoperative prediction of *pTERT* mutation status in GBM.

FLAIR hyperintensity is an important imaging feature for the assessment of GBM. Previous studies have shown that increased FLAIR signal may serve as an early indicator of glioma progression and correlate with poor prognosis (Zhang, Zhou, et al. 2024). Additionally, FLAIR‐based imaging features have demonstrated diagnostic value in predicting molecular subtypes of glioma. For example, the T2/FLAIR mismatch sign is a well‐recognized imaging marker for identifying *IDH*‐mutant astrocytomas. Although previous work has suggested that *pTERTm* GBM is more likely to exhibit necrotic hypointensity on FLAIR and predilection for frontotemporal regions, the lack of quantitative thresholds limits the clinical utility of these findings. Recently, radiomics and deep learning analyses based on FLAIR sequences have been applied to predict molecular alterations such as *IDH* mutation, *1p/19q* co‐deletion, and *MGMT* methylation (Koska and Koska 2025). However, these methods require specialized software and are difficult to translate into routine clinical practice. In contrast, our study utilized subregional segmentation based on conventional fusion imaging and open‐source tools, providing a more accessible approach for exploring the relationship between FLAIR SI and *pTERT* mutation.

Subregional quantitative analysis of FLAIR signal provided added value in predicting *pTERT* phenotype. We observed significant differences in the FLAIR metrics of both the enhancing tumor core and the non‐enhancing edema region between *pTERTm* and *pTERTw* GBM. Metrics from the enhancement region demonstrated the highest discriminative ability, consistent with the biological characteristics of *pTERTm* GBM, which tends to exhibit increased cellular proliferation, reduced extracellular space, and abundant neovascularization, features that manifest as increased signal heterogeneity on FLAIR imaging (Kamimura et al. 2024). Elevated FLAIR_SD_ and rFLAIR in the edema region of *pTERTm* GBM may be related to higher levels of inflammatory cytokines such as interleukin‐6 and tumor necrosis factor, which contribute to extensive peritumoral edema (Mosrati et al. 2015). However, because vasogenic edema dominates the non‐enhancing region and tumor cell infiltration is limited, edema‐related metrics had relatively lower predictive value compared with enhancement‐derived metrics. Importantly, whole‐lesion FLAIR metrics did not differ significantly between phenotypes, underscoring the limitation of evaluating the tumor as a single region of interest. This aligns with radiomics research demonstrating superior performance of subregional models in predicting molecular features of glioma (Behling et al. 2022; Zhang, Zhou, et al. 2024; Moon et al. 2024).

No significant associations were observed between *pTERT* phenotype and MRI morphological signs such as tumor location or involvement of the ependyma or corpus callosum. Although earlier studies reported that *pTERTm* gliomas demonstrate certain locational preferences (Fan et al. 2016; Tang et al. 2017; Powter et al. 2021), our findings did not reproduce this pattern, likely due to limited sample size. Larger studies are needed to evaluate whether tumor location consistently correlates with *pTERT* status.

Regarding functional MRI parameters, *pTERTm* tumors exhibited lower rADC values than *pTERTw* tumors, suggesting higher cellularity, more prominent angiogenesis, and intensified inflammatory response. This differs from the findings of Yamashita et al. (2019), who reported no significant ADC differences; however, their study used mean ADC, whereas we used minimum ADC, which may better reflect tumor cell density and diffusion restriction. In MRS, the higher Cho/Cr ratio observed in *pTERTm* GBM is consistent with previous results (Tian et al. 2020) and reflects increased membrane turnover and proliferative activity. Because functional MRI data were not available for all patients, these variables were not included in the final prediction model but remain of biological interest.

Among clinical characteristics, age was significantly higher in the *pTERTm* group, consistent with prior findings (Priya et al. 2021). Therefore, age was incorporated into the prediction model and contributed meaningfully to diagnostic performance. The resulting nomogram, which integrated clinical and subregional imaging features, provided individualized estimates of the probability of *pTERT* mutation and may hold practical value for preoperative patient assessment.

Although the STUPP protocol remains the standard of care for GBM, survival outcomes have shown little improvement over the past decade. Emerging targeted drug delivery systems, nanoformulated chemotherapeutics, and nuclear medicine–based approaches (including radionanoparticles, radiopeptides, and radioimmunotherapy) offer promising treatment avenues (Ganau 2014; Bailly et al. 2019). Given that *pTERT* mutation is associated with therapy resistance and early recurrence, integrating imaging‐derived subregional features with molecular phenotyping may help guide these precision‐based therapeutic strategies. Specifically, identifying high‐risk subregions within individual tumors may facilitate targeted delivery of nanodrugs or radionuclide therapies to areas with the greatest recurrence potential, thereby enhancing treatment efficacy while minimizing collateral damage.

Several limitations should be acknowledged. First, this study was retrospective and had a relatively small sample size, which may introduce selection bias and limit statistical power. Although we applied LOOCV to maximize data utilization and enhance internal stability, and further evaluated the model in an independent validation cohort, larger multicenter datasets will be necessary to fully confirm the generalizability of our findings. Second, pathological validation corresponding to individual subregions was unavailable; stereotactic biopsies could help confirm the histopathological basis of quantitative imaging metrics. Third, only a limited number of variables were included in the final model due to the constraints of conventional imaging. Future work should incorporate advanced functional MR techniques and emerging quantitative imaging biomarkers to further enhance the predictive value of *pTERT* mutation models.

## 5 Conclusion

In summary, specific quantitative FLAIR‐based metrics extracted from tumor subregions were independent predictors of *pTERT* mutation in patients with GBM. Subregional segmentation of CE T1WI–FLAIR fusion images provided a more accurate representation of tumor heterogeneity than whole‐lesion analysis and improved discrimination between *pTERTm* and *pTERTw* phenotypes. The integration of fusion‐image‐derived quantitative metrics with clinical variables enabled the construction of a reliable predictive model for *pTERT* mutation. Future studies with larger sample sizes and multimodal functional imaging data are needed to further enhance individualized prediction and support the development of precision imaging–genomic tools for GBM phenotype assessment.

## Author Contributions

B.G., G.Q., and T.Y. have contributed substantially to the concept and design of the article; the acquisition, analysis, and interpretation of data; and the drafting of the article. G.Q., Y.L., and T.Y. revised the article critically for important intellectual content. B.G. and G.Q. collected the data of all patients and made an analysis and interpretation of the data for the article. B.G. and T.Y. have agreed to be accountable for all aspects of the work in ensuring that questions related to the accuracy or integrity of any part of the work are appropriately investigated and resolved. All authors contributed to the article and approved the submitted version.

## Funding

This work was supported by Hebei Provincial Natural Science Foundation (H2025206451) and Key project plan of medical scientific research in Hebei province (20221104).

## Ethics Statement

This retrospective study was approved by the ethics committee of The Second Hospital of Hebei Medical University (2024‐R186). All procedures performed in studies involving human participants were in accordance with the ethical standards of the institutional and/or national research committee and with the 1964 Helsinki Declaration and its later amendments or comparable ethical standards.

## Consent

Informed consent was obtained from all subjects.

## Conflicts of Interest

The authors declare no conflicts of interest.

## Data Availability

The data that support the findings of this study are available from the corresponding author upon reasonable request.

## References

[brb371284-bib-0001] Bailly, C. , A. Vidal , C. Bonnemaire , et al. 2019. “Potential for Nuclear Medicine Therapy for Glioblastoma Treatment.” Frontiers in Pharmacology 10: 772. 10.3389/fphar.2019.00772. 31354487 PMC6637301

[brb371284-bib-0002] Behling, F. , J. Rang , E. Dangel , et al. 2022. “Complete and Incomplete Resection for Progressive Glioblastoma Prolongs Post‐Progression Survival.” Frontiers in Oncology 12: 755430. 10.3389/fonc.2022.755430. 35251956 PMC8888692

[brb371284-bib-0004] Chen, L. , R. Chen , T. Li , et al. 2023. “MRI Radiomics Model for Predicting TERT Promoter Mutation Status in Glioblastoma.” Brain and Behavior 13: e3324. 10.1002/brb3.3324. 38054695 PMC10726789

[brb371284-bib-0005] Fan, X. , Y. Wang , Y. Liu , et al. 2016. “Brain Regions Associated With Telomerase Reverse Transcriptase Promoter Mutations in Primary Glioblastomas.” Journal of Neuro‐Oncology 128: 455–462. 10.1007/s11060-016-2132-y. 27230769

[brb371284-bib-0006] Ganau, M. 2014. “Tackling Gliomas With Nanoformulated Antineoplastic Drugs: Suitability of Hyaluronic Acid Nanoparticles.” Clinical & Translational Oncology 16: 220–223. 10.1007/s12094-013-1114-1. 24072561

[brb371284-bib-0007] Gao, L. , T. Yuan , Y. Liu , X. Yang , Y. Li , and G. Quan . 2024. “Prognostic Nomogram Model Based on Quantitative Metrics of Subregions Surrounding Residual Cavity in Glioblastoma Patients.” Journal of Cancer Research and Clinical Oncology 150: 483. 10.1007/s00432-024-06008-6. 39487262 PMC11530551

[brb371284-bib-0008] Ivanidze, J. , M. Lum , D. Pisapia , et al. 2019. “MRI Features Associated With TERT Promoter Mutation Status in Glioblastoma.” Journal of Neuroimaging 29: 357–363. 10.1111/jon.12596. 30644143

[brb371284-bib-0009] Kamimura, S. , Y. Mitobe , K. Nakamura , et al. 2024. “Association of ADC of Hyperintense Lesions on FLAIR Images With TERT Promoter Mutation Status in Glioblastoma IDH Wild Type.” Surgical Neurology International 15: 108. 10.25259/SNI_63_2024. 38628517 PMC11021064

[brb371284-bib-0010] Koska, İ. Ö. , and Ç. Koska . 2025. “Deep Learning Classification of MGMT Status of Glioblastomas Using Multiparametric MRI With a Novel Domain Knowledge Augmented Mask Fusion Approach.” Scientific Reports 15: 3273. 10.1038/s41598-025-87803-0. 39863759 PMC11762293

[brb371284-bib-0011] Kurokawa, R. , M. Kurokawa , A. Baba , et al. 2022. “Major Changes in 2021 *World Health Organization Classification of Central Nervous System Tumors* .” Radiographics 42: 1474–1493. 10.1148/rg.210236. 35802502

[brb371284-bib-0012] Liu, S. , Y. Zhang , Z. Kong , et al. 2022. “Feasibility of Evaluating the Histologic and Genetic Subtypes of WHO Grade II–IV Gliomas by Diffusion‐Weighted Imaging.” BMC Neuroscience 23: 72. 10.1186/s12868-022-00750-8. 36471242 PMC9720933

[brb371284-bib-0013] Moon, H. H. , J. Jeong , J. E. Park , et al. 2024. “Generative AI in Glioma: Ensuring Diversity in Training Image Phenotypes to Improve Diagnostic Performance for IDH Mutation Prediction.” Neuro‐Oncology 26: 1124–1135. 10.1093/neuonc/noae012. 38253989 PMC11145451

[brb371284-bib-0014] Mosrati, M. A. , A. Malmström , M. Lysiak , et al. 2015. “TERT Promoter Mutations and Polymorphisms as Prognostic Factors in Primary Glioblastoma.” Oncotarget 6: 16663–16673. 10.18632/oncotarget.4389. 26143636 PMC4599297

[brb371284-bib-0015] Murugan, A. K. , S. Kannan , and A. S. Alzahrani . 2025. “TERT Promoter Mutations in Gliomas: Molecular Roles in Tumorigenesis, Metastasis, Diagnosis, Prognosis, Therapeutic Targeting, and Drug Resistance.” Biochimica et Biophysica Acta ‐ Reviews on Cancer 1880: 189243. 10.1016/j.bbcan.2024.189243. 39674418

[brb371284-bib-0016] Park, Y. W. , S. S. Ahn , C. J. Park , et al. 2020. “Diffusion and Perfusion MRI May Predict EGFR Amplification and the TERT Promoter Mutation Status of IDH‐Wildtype Lower‐Grade Gliomas.” European Radiology 30: 6475–6484. 10.1007/s00330-020-07090-3. 32785770

[brb371284-bib-0017] Powter, B. , S. A. Jeffreys , H. Sareen , et al. 2021. “Human TERT Promoter Mutations as a Prognostic Biomarker in Glioma.” Journal of Cancer Research and Clinical Oncology 147: 1007–1017. 10.1007/s00432-021-03536-3. 33547950 PMC7954705

[brb371284-bib-0018] Priya, S. , C. Ward , T. Locke , et al. 2021. “Glioblastoma and Primary Central Nervous System Lymphoma: Differentiation Using MRI Derived First‐Order Texture Analysis—A Machine Learning Study.” Neuroradiology Journal 34: 320–328. 10.1177/1971400921998979. 33657924 PMC8447821

[brb371284-bib-0020] Tang, F. , X. Chen , J. S. Liu , et al. 2023. “TERT Mutations‐Associated Alterations in Clinical Characteristics, Immune Environment and Therapy Response in Glioblastomas.” Discover Oncology 14: 148. 10.1007/s12672-023-00760-w. 37566174 PMC10421840

[brb371284-bib-0021] Tang, Q. , Y. Lian , J. Yu , Y. Wang , Z. Shi , and L. Chen . 2017. “Anatomic Mapping of Molecular Subtypes in Diffuse Glioma.” BMC Neurology 17: 183. 10.1186/s12883-017-0961-8. 28915860 PMC5602933

[brb371284-bib-0022] Tian, H. , H. Wu , G. Wu , and G. Xu . 2020. “Noninvasive Prediction of TERT Promoter Mutations in High‐Grade Glioma by Radiomics Analysis Based on Multiparameter MRI.” BioMed Research International 2020: 3872314. 10.1155/2020/3872314. 32509858 PMC7245686

[brb371284-bib-0023] Yamashita, K. , R. Hatae , A. Hiwatashi , et al. 2019. “Predicting TERT Promoter Mutation Using MR Images in Patients With Wild‐Type IDH1 Glioblastoma.” Diagnostic and Interventional Imaging 100: 411–419. 10.1016/j.diii.2019.02.010. 30948344

[brb371284-bib-0024] Yuan, T. , Z. Gao , F. Wang , et al. 2022. “Relative T2‐FLAIR Signal Intensity Surrounding Residual Cavity Is Associated With Survival Prognosis in Patients With Lower‐Grade Gliomas.” Frontiers in Oncology 12: 960917. 10.3389/fonc.2022.960917. 36185187 PMC9520477

[brb371284-bib-0025] Zhang, H. , Y. Ouyang , H. Zhang , et al. 2024. “Sub‐Region Based Radiomics Analysis for Prediction of Isocitrate Dehydrogenase and Telomerase Reverse Transcriptase Promoter Mutations in Diffuse Gliomas.” Clinical Radiology 79: e682–e691. 10.1016/j.crad.2024.01.030. 38402087

[brb371284-bib-0026] Zhang, H. , H. Zhang , Y. Zhang , et al. 2023. “Deep Learning Radiomics for the Assessment of Telomerase Reverse Transcriptase Promoter Mutation Status in Patients With Glioblastoma Using Multiparametric MRI.” Journal of Magnetic Resonance Imaging 58: 1441–1451. 10.1002/jmri.28671. 36896953

[brb371284-bib-0027] Zhang, H. , B. Zhou , H. Zhang , Y. Zhang , Y. Lei , and B. Huang . 2024. “Peritumoral Radiomics for Identification of Telomerase Reverse Transcriptase Promoter Mutation in Patients With Glioblastoma Based on Preoperative MRI.” Canadian Association of Radiologists Journal 75: 143–152. 10.1177/08465371231183309. 37552107

[brb371284-bib-0028] Zhang, H. W. , G. W. Lyu , W. J. He , et al. 2020. “DSC and DCE Histogram Analyses of Glioma Biomarkers, Including IDH, MGMT, and TERT, on Differentiation and Survival.” Academic Radiology 27: e263–e271. 10.1016/j.acra.2019.12.010. 31983532

